# Neutropenia after the coadministration of clozapine and nirmatrelvir-ritonavir in a patient with SARS-CoV-2 infection: A case report with a literature review

**DOI:** 10.3389/fpsyt.2022.1096006

**Published:** 2022-12-21

**Authors:** Chun-I Liu, Kah Kheng Goh, Chun-Hsin Chen

**Affiliations:** ^1^Department of Psychiatry, Wan Fang Hospital, Taipei Medical University, Taipei, Taiwan; ^2^Department of Psychiatry, School of Medicine, College of Medicine, Taipei Medical University, Taipei, Taiwan; ^3^Psychiatric Research Center, Wan Fang Hospital, Taipei Medical University, Taipei, Taiwan

**Keywords:** neutropenia, clozapine, SARS-CoV-2, drug-related side effects and adverse reactions, drug interactions

## Abstract

**Background:**

Schizophrenia is considered one of the major risk factors for mortality from SARS-CoV-2 infection. Early antiviral treatment is important to decrease the risk of mortality. Currently, Paxlovid (nirmatrelvir-ritonavir) has been widely used in SARS-CoV-2 patients with risk factors. However, drug–drug interactions with anti-psychotics are prominent and complicated.

**Case presentation:**

We report a clozapine-treated patient with SARS-CoV-2 infection who developed neutropenia after coadministration with Paxlovid. In this case, clozapine was used for over 15 years, without neutropenia development. However, severe neutropenia (absolute neutrophil count = 523/μl) developed 3 days after the coadministration of Paxlovid 2 doses per day, valproic acid 1,000 mg per day and clozapine 100 mg per day. The development of neutropenia may be attributed to the complicated interaction among Paxlovid, SARS-CoV-2 infection, valproic acid, fluvoxamine and clozapine.

**Conclusions:**

Neutropenia is a rare but life-threatening event if a concomitant infection occurs. The risk may increase during SARS-CoV-2 infection and the coadministration of clozapine and Paxlovid. Although the exact causes of neutropenia in this patient are not fully clear, the white blood cell count and absolute neutrophil count should be closely monitored during the administration of Paxlovid in clozapine-treated patients with SARS-CoV-2 infection.

## Background

Antiviral treatment has been important since the outbreak of the SARS-CoV-2 pandemic. Nirmatrelvir-ritonavir (Paxlovid) is considered the most effective antiviral regimen. Compared to remdesivir and molnupiravir, Paxlovid can prevent more SARS-CoV-2 patients with risk factors from requiring hospitalization and was the first-line antiviral medication for SARS-CoV-2 until June 2022 (1). Because of the cytochrome P450 (CYP) 3A4, 1A2, and possibly 2D6 inhibition effect of ritonavir, drug–drug interactions should be considered when prescribing Paxlovid (2).

Schizophrenia is the second highest risk factor for dying from SARS-CoV-2 infection (3). Early administration of antiviral agents is essential for patients with schizophrenia who are infected by SARS-CoV-2. Currently, clozapine is broadly used in treatment-refractory patients with schizophrenia. *In vitro* and *in vivo* studies show that CYP3A4 and CYP1A2 are the major metabolic pathways, but CYP2D6 plays a minor role of clozapine metabolism (4, 5). Additionally, the CYP metabolic pathway may be affected by medications that inhibit or induce CYP. Thus far, the coadministration of clozapine with Paxlovid is contraindicated due to potential CYP1A2 inhibition. To date, there is no clinical report about the adverse effects of the coadministration of clozapine and Paxlovid in patients with active SARS-CoV-2 infection. Here, we present a patient who developed isolated neutropenia after the coadministration of clozapine and Paxlovid during active SARS-CoV-2 infection.

## Case presentation

Ms. L was a 41-year-old Taiwanese Han female patient with schizophrenia. She had no known underlying physical disease, with normal blood sugar, blood pressure, serum low-density lipoprotein, total cholesterol and triacylglycerol levels. Her first psychotic symptoms occurred at the age of 20 years, with the manifestation of delusions of grandeur and religion, disorganized behaviors, and negative symptoms, including avolition and flat affect. She had several manic episodes with the presentation of expansive mood, hypertalkativeness, multiple goal-oriented behaviors, and flight of ideas. She was repeatedly admitted to the psychiatric ward due to psychotic or manic-like symptoms (15 times from 2007 to 2022) and had used clozapine since 2007. Although we did not measure blood level of clozapine, she even received fluvoxamine to increase clozapine blood levels and optimize the serum clozapine to norclozapine ratio in 2010. The clozapine dose ranged from 200 to 500 mg per day, and valproic acid and other antipsychotics (haloperidol and paliperidone) were sometimes used for augmentation due to poor response to clozapine. During the use of clozapine, the white blood cell (WBC) count ranged from 3,100 to 13,800/μl, and the absolute neutrophil count (ANC) ranged from 1,499 to 11,422/μl before April 10, 2022. The timing of blood sample, which depended on her appointments, was not fixed. On April 11, 2022, because she did not take medications regularly, the patient was admitted to the acute psychiatric ward due to psychotic exacerbation.

The patient's symptoms at the time of hospitalization included delusions of grandiosity and persecution, hypertalkativeness, elevated mood, irritability, decreased sleep needs, and increased verbal aggression toward her family. Medications at the time of hospitalization included paliperidone palmitate 525 mg every 3 months and valproic acid 1,000 mg and clozapine 200 mg daily. The blood profiles on April 11 showed a WBC count of 5,080 cells/μL and an ANC of 3,434 cells/μL. Since the patient missed taking clozapine and valproic acid for weeks, we restarted clozapine from 25 to 275 mg daily in the following 8 weeks. To elevate the blood level of clozapine, fluvoxamine maleate 50 mg daily was added on May 10, 2022. The WBC count and ANC were 6,170 and 2,795 cells/μL, respectively, on May 16 (the 6th day after adding fluvoxamine). On June 1, she developed a dry cough, and her body temperature rose to 39.0 degrees Celsius. The patient's hemogram revealed that her blood c-reactive protein level was 0.9 mg/dL, her WBC count was 4,450 cells/μL, and her ANC was 2,474 cells/μL. The results of her SARS-CoV-2 reverse transcription-polymerase chain reaction (RT–PCR) test on June 1 were positive, with a cycle threshold (ct) value of 12.5. She received one dose of Paxlovid (ritonavir 100 mg and nirmatrelvir 150 mg) every 12 h from June 2 to June 7 for acute SARS-CoV-2 infection. In total, 10 doses of Paxlovid were given. From the first day and the last day, she was given one dose to maintain an adequate prescription interval. To reduce the drug–drug interaction, her clozapine was tapered off to 175 mg on June 2 and then to 100 mg on June 3. Fluvoxamine was discontinued, and valproic acid was tapered off to 1,000 mg on June 2. On the routine blood test on June 5 (the third day after the coadministration of Paxlovid and clozapine), the WBC count and ANC were reduced to 3,490 and 523 cells/μL, respectively. We discontinued clozapine immediately, and valproic acid was tapered off to 500 mg on June 6. She did not have any upper respiratory tract symptoms, fever, or other signs of infection. Her blood clozapine level was not obtained. The patient's repeated SARS-CoV-2 RT–PCR tests showed Ct values of 25.0 on June 5 and 28.1 on June 8. The follow-up WBC count and ANC were 4,170 and 1,547 cells/μL, respectively, on June 8. Because the patient's agitation and delusions of grandiosity worsened, 100 mg per day of clozapine was reintroduced on June 9. On June 12, she had negative SARS-CoV-2 RT–PCR findings. The WBC count and ANC were 5,120 and 2,468 cells/μL, respectively, on June 13. We gradually titrated clozapine to 150 mg per day and added 25 mg fluvoxamine per day to stabilize the exacerbated psychotic symptoms and agitative status. Table 1 summarizes the patient's medications and laboratory data from May 30 to June 23.

**Table 1:** Summary of medications and laboratory data before and after SARS-CoV-2 infection.

	**5/30**	**6/1**	**6/2**	**6/3**	**6/4**	**6/5**	**6/6**	**6/7**	**6/8**	**6/13**	**6/20**	**6/26**
SARS-CoV-2 CT value	–	12.5	–	–	–	25.0	–	–	28.1	Negative	–	–
WBC count per uL	8,630	4,450	–	–	–	3,490	–	–	4,170	5,120	4,210	6,720
ANC per uL	6,300	2,474	–	–	–	523	–	–	1,547	2,468	1,372	3,884
Serum valproic acid level, ug/mL	–	–	–	–	–	62.0	–	–	–	–	65.3	82.2
Paxlovid dosage	0	0	1	2	2	2	2	1	0	0	0	0
Clozapine dosage, mg per day	250	275	175	100	100	0	0	0	0	125	150	150
Fluvoxamine dosage, mg per day	50	50	0	0	0	0	0	0	0	0	25	25
Valproic acid dosage, mg per day	1,500	1,500	1,000	1,000	1,000	1,000	500	500	500	1,000	1,200	1,200

–, not tested; ANC, absolute neutrophil count; CT, cycle threshold; WBC, white blood cell.

## Discussion and conclusions

Severe neutropenia with concomitant infections is a fatal complication that may be induced by immune-compromised status, immunosuppressive agents and even the prolonged use of antiviral therapy (6). Regarding the polypharmacy status and the comorbidity of SARS-CoV-2 infection, this unique case permits further discussion of the potential etiologies of neutropenia.

First, the initiation and titration of clozapine was reported to be associated with new-onset neutropenia. The pathogenetic mechanism of clozapine-related agranulocytosis was hypothesized, with clozapine potentially inducing antibodies against neutrophils, but the category and the type of antibody remained unclear (7). A current meta-analysis pointed out that neutropenia associated with clozapine occurs early with a substantial decline in risk after 1 year of exposure (8). Because our patient had used clozapine for nearly 15 years and we gradually titrated the clozapine dose, no neutropenia was noted before the current SARS-CoV-2 infection. Therefore, neutropenia due to clozapine alone is less likely.

Second, not only clozapine but also SARS-CoV-2 infection causes significant changes in the WBC count (9). Although neutropenia has been reported in post-COVID-19 cases, it is rare in cases of SARS-CoV-2 active infection without superimposed bacterial infection or malignancies (10–12). However, severe granulocytopenia has been reported in SARS-CoV-2-infected patients with the long-term use of clozapine (13, 14). A retrospective study also concluded that in patients treated with clozapine, the ANC, WBC count and lymphocyte count significantly decreased between baseline and the first 7 days of SARS-CoV-2 infection (15). Previous studies indicated that the decreased ANC is temporary (16). It rebounded to baseline in the second week even without the reduction of the clozapine dosage. Although the ANC is decreased, recent case series have illustrated the safe use of clozapine during SARS-CoV-2 infection (17). Smits et al. recommended the continuation of clozapine in SARS-CoV-2-infected patients even when they developed neutropenia (18). Therefore, we suggest that the neutropenia that occurred in our patient may be due to SARS-CoV-2 infection.

Third, neutropenia has been reported in human immunodeficiency virus (HIV)-infected patients with viral loads < 20 copies/mL who were treated with clozapine and ritonavir previously (19), suggesting that the potential casual effect of neutropenia and the combination of ritonavir and clozapine. Ritonavir (a component of Paxlovid) is another inhibitor of both CYP1A2 and CYP3A4, which may increase blood level of clozapine. As we mentioned previously, Paxlovid is not recommended in clozapine-treated patients due to possible drug–drug interactions. Acute viral infection may increase blood level of clozapine in this patient (20, 21). One possible explanation could be that elevated α-1-acid glycoprotein concentrations during inflammation increase plasma clozapine-α-1-acid glycoprotein binding, results in elevated total clozapine plasma concentrations (22). In addition, concurrent use of valproate (23) and fluvoxamine (24, 25), has also been reported to elevate blood level of clozapine. However, no consensus exists to explain whether elevated clozapine levels increase the risk of neutropenia currently. We suggest that the coadministration of psychotropics and Paxlovid is one of the possible causes of neutropenia.

Fourth, valproate causes bone marrow suppression, which can be associated with idiosyncratic neutropenia (26). Although our patient had used valproate in the past, she did not develop neutropenia, and neutropenia due to valproate use alone is less likely.

Fifth, current evidence could not fully elucidate the causal relationship among Paxlovid, fluvoxamine and neutropenia. However, no report exists to describe whether fluvoxamine and Paxlovid may cause neutropenia. As a newly introduced medication, the adverse events caused by Paxlovid may need more investigation.

The readers are warned not to overinterpret the findings described for this patient because this study has several limitations: (1) As a case report, the findings cannot be generalized. More investigation should be performed to illustrate the complicated interaction among clozapine, Paxlovid and SARS-CoV-2 infection. (2) Serum clozapine levels were not analyzed in this patient. We do not know the changes in blood clozapine levels after Paxlovid use. The further relationship between the clozapine level and neutropenia cannot be proven.

In conclusion, neutropenia is a rare but potentially life-threatening event. The risk of neutropenia may increase during SARS-CoV-2 infection. The coadministration of Paxlovid may also contribute to the risk of neutropenia in this patient. Although the exact causes of neutropenia in this patient are not fully clear, the WBC count and ANC should be closely monitored during the administration of Paxlovid in clozapine-treated patients with SARS-CoV-2 infection.

## Data availability statement

The original contributions presented in the study are included in the article/supplementary material, further inquiries can be directed to the corresponding author/s.

## Ethics statement

The studies involving human participants were reviewed and approved by Taipei Medical University-Joint Institutional Review No. N202207057. The patients/participants provided their written informed consent to participate in this study.

## Author contributions

C-IL reviewed the case and wrote the manuscript. KG contributed to the case. C-HC revised the manuscript. All authors have read and approved the manuscript.
